# Comparison of Five-Day vs. Fourteen-Day Incubation of Cultures for Diagnosis of Periprosthetic Joint Infection in Hip Arthroplasty

**DOI:** 10.3390/jcm13154467

**Published:** 2024-07-30

**Authors:** Catalina Baez, Robert MacDonell, Abtahi Tishad, Hernan A. Prieto, Emilie N. Miley, Justin T. Deen, Chancellor F. Gray, Hari K. Parvataneni, Luis Pulido

**Affiliations:** 1 Department of Orthopaedic Surgery and Sports Medicine, University of Florida, Gainesville, FL 32607, USA; rtmacdonell@gmail.com (R.M.); prieth@ortho.ufl.edu (H.A.P.); emilie.miley1@gmail.com (E.N.M.); 2 College of Medicine, University of Florida, Gainesville, FL 32607, USA; tishad100@ufl.edu; 3 Florida Orthopaedic Institute, Gainesville, FL 32607, USA; deenju812@gmail.com (J.T.D.); chancellor.gray@gmail.com (C.F.G.); hkparvataneni@gmail.com (H.K.P.)

**Keywords:** prosthetic joint infection, total hip arthroplasty, intraoperative cultures, culture hold times

## Abstract

**Background:** Periprosthetic joint infections (PJI) are among the most morbid complications in total hip arthroplasty (THA). The ideal incubation time, however, for intraoperative cultures for PJI diagnosis remains unclear. As such, the aim of this study was to determine if any differences existed in culture-positive rates and organism detection between five-day and fourteen-day cultures. **Methods:** This retrospective cohort study consisted of THA cases diagnosed with PJI performed between May 2014 and May 2020 at a single tertiary-care institution. Analyses compared five-day and fourteen-day cultures and carried out a pre-specified subgroup analysis by organism and PJI type. **Results:** A total of 147 surgeries were performed in 101 patients (57.1% females), of which 65% (n = 98) obtained five-day cultures and 34% (n = 49) obtained fourteen-day cultures. The positive culture rate was 67.3% (n = 99) with *Staphylococcus aureus* being the most common pathogen identified (n = 41 specimens, 41.4%). The positive culture rate was not significantly different between groups (66.3% five-day, 69.4% fourteen-day, *p* = 0.852). Fourteen-day cultures had a significantly longer time-to-positive culture (5.0 days) than five-day cultures (3.0 days, *p* < 0.001), a higher rate of fungi (5.6% vs. 0%), and a lower rate of Gram-negatives (4.5% vs. 18.7%, *p* = 0.016). **Conclusions:** Fourteen-day cultures did not increase the positivity rate, had higher rates of slow-growth pathogens, and had a longer time-to-positivization than five-day cultures. Prolonged culture holds may provide more thorough organism detection for PJI without increasing the diagnostic culture yield.

## 1. Introduction

Prosthetic joint infections (PJI) have a seemingly sparse incidence of 0.76% to 1.24% [1]. However, they are associated with significant morbidity, causing severe psychological [2] and economic [3] burden on the patient, including mortality, with death rates increasing progressively following revision arthroplasty from 5.5 to 8% (1-year postoperative), 11 to 25% (2-years postoperative), and 40 to 45% (5-years postoperative) [4,5,6,7]. In addition, there has been a 2.6-fold increase in annual procedures performed for PJI treatment between 2006 and 2013 [8]. The yearly cost of these procedures exceeded USD 500 million in 2017 and is expected to increase to USD 753.4 million by 2030 [9] as the annual volume of total hip arthroplasty (THA) surgery will increase by 174% by 2030 [1]. This historical increase in the incidence of PJI cases highlights the need for clinicians and researchers to determine the best approach to diagnose PJI.

Released in 2018, the revised Musculoskeletal Infection Society (MSIS) criteria uses a scoring system based on a combination of culture, laboratory, and clinical presentation findings to yield a diagnosis with 99.7% sensitivity and 99.5% specificity [10]. A major criterion for diagnosis is the isolation of a single organism in two separate cultures of periprosthetic tissue or synovial fluid [10]. However, a lack of literature exists to determine the ideal culture time for periprosthetic tissue samples as it appears highly organism-dependent [11,12,13,14,15] with wide variation in incubation times for anaerobic and aerobic samples [16]. A 2018 study demonstrated that while most infected samples yielded positive culture results within five days, some samples (i.e., *Cutibacterium acnes* (*C. acnes*)) needed ten or more days to yield positive culture results [11]. Previous PJI diagnosis and management guidelines have failed to address recommendations on the ideal culture incubation duration [10,17,18], and historical trends have proposed culture incubation durations varying from four to fourteen days [12,17].

Considering these inconsistencies in the literature, the aim of this study was to determine if there was a difference in positivity rates, organism growth, time of growth, treatment, and disease-free survival between five-day and fourteen-day cultures in THA PJI. Additionally, we sought to determine the organism profile for PJI in our tertiary-care institution and whether there is an association between preoperative Synovasure^®^ (Zimmer Biomet, Warsaw, IN, USA) synovial fluid cultures and intraoperative culture organism characterization performed at our hospital laboratory.

## 2. Materials and Methods

After obtaining Institutional Review Board approval, a retrospective cohort study was performed on THA patients diagnosed with PJI from May 2014 through May 2020. Our hospital laboratory transitioned culture hold times in May 2018. Before this time, all cultures were held for five days. Following this transition, cultures were held for fourteen days. All findings from this study are reported following the Strengthening the Reporting of Observational Studies in Epidemiology (STROBE) guidelines [19].

### 2.1. Participants

The inclusion criteria were THA cases with a diagnosis and treatment for PJI that underwent revision surgery and intraoperative cultures were obtained (CPT codes: 27134, 27137, 27138, 27091, 20680, 11981, 20702, 10061). We included only patients with a minimum one-year follow-up visit. The diagnosis of PJI was based on the 2018 revised MSIS criteria [10]. We excluded all cases where PJI diagnostic criteria were not met, subjects for which intraoperative cultures were not obtained, and patients with less than one-year follow-up.

### 2.2. Variables and Outcome Measures

Demographic data, including patient medical history (i.e., age, body mass index (BMI), sex), comorbidities (i.e., smoking status, diabetes mellitus (DM)), and PJI data were collected for this study via chart review of the electronic medical record. In addition, perioperative, operative, and postoperative data, surgical and antibiotic treatment, and complications reported during the first year following surgery were collected. Given that the culture hold time was the same for all specimens obtained during each surgery, we compared outcomes across five-day and fourteen-day culture holds by grouping all intraoperative culture specimens into one surgical culture sample (i.e., if one or more of the specimens obtained intraoperatively were positive, this surgical case was determined to be a culture-positive case) for statistical analysis. All organism-specific data were compared using individual culture specimen data for those that reported microbial growth. Thus, the number of intraoperative specimens sent in for culture, the number of specimens that were positive for microbial growth, the time to positive culture, organisms reported, antibiotic selection, definitive surgical intervention (i.e., debridement, antibiotics and implant retention (DAIR), one-stage revision, two-stage revision, resection arthroplasty, and amputation), surgical treatment failure, and disease-free survival were compared using the grouped surgical culture sample data. We defined surgical treatment failure according to the MSIS 2018 consensus [20], where Tiers 3 and 4 represent surgical treatment failure. Disease-free survival was defined as the total follow-up time without further PJI management. 

The time to positive culture by organism, type of PJI (i.e., early postoperative, acute hematogenous, and chronic), surgical treatment failure, and complications during the first year after surgery (i.e., readmissions in the first 30 days postoperative and death in the first 365 days postoperative) were obtained to define the PJI organism profile for this institution. Finally, Synovasure^®^ culture reports were collected to compare with intraoperative culture organisms.

### 2.3. Statistical Analysis

Statistical analyses were performed using the statistical software IBM SPSS Version 28. Categorical measures were summarized as counts (n) and percentages (%), and continuous measures were summarized as means and standard deviations (SD). Categorical variables were analyzed for between-group comparisons with chi-square and Fisher’s exact tests. Given the non-normal characteristics of the data, the Kruskal–Wallis test was used to compare continuous data. Survival analyses were performed using Kaplan–Meier curves and the log-rank test for between-group comparisons. Prespecified subgroup analyses were completed for low-virulence organisms (i.e., *C. acnes*, *Cutibacterium albicans* (*C. albicans*), mycobacteria, and anaerobes) and different organism types (i.e., Gram-positive, Gram-negative, anaerobes, fungi). Disease-free survival was defined as the total follow-up time without further PJI management, and case censoring was defined as surgical treatment failure according to the MSIS 2018 consensus [10]. Statistical significance was predefined at a *p* < 0.05.

## 3. Results

A total of 147 surgeries were identified for 101 patients with a PJI diagnosis from May 2014 to May 2020. Of these, 66.7% (n = 98) of the surgical culture samples were held for five days, and 33.3% (n = 49) were held for fourteen days. In total, 57.1% (n = 84) were male, and 63% (n = 42.9) were female, with a mean age of 62.0 (±12.7) years and a mean BMI of 30.8 (±9.0). A total of 19% (n = 28) of the patients in the sample reported being active smokers at the time of PJI diagnosis, and 32% (n = 47) had a diagnosis of diabetes mellitus (DM). The McPherson host grade [21] rates were 12.2% (n = 18) grade A, 46.3% (n = 68) grade B, and 41.5% (n = 61) grade C. Additionally, 71.4% (n = 105) of cases were classified as chronic PJI, 15.6% (n = 23) as acute hematogenous, and 12.9% (n = 19) as early postoperative. No significant differences were identified for patient demographics between the five-day and fourteen-day groups (Table 1).

### 3.1. Comparison of Five-Day and Fourteen-Day Cultures

The culture-positive rate for the full cohort was 67.3% (n = 99), with no significant differences between five-day and fourteen-day cultures (*p* = 0.852, Table 2). The mean number of individual specimens obtained during each surgery was 8.0 (±4.2), of which a mean of 2.5 (±1.4) specimens grew detectable organisms. There was no significant differences between five-day and fourteen-day cultures for the total number of specimens sent for culture (*p* = 0.079) or the number of positive specimens (*p* = 0.527, Table 2).

The median time to positive culture was 4.0 days (range = 0–23 days). The time to positive culture was significantly longer for fourteen-day cultures with a median time of 5.0 days (range = 0–23 days), whereas the five-day cultures had a median of 3.0 days (range = 0–7 days, *p* < 0.001; Table 2). No significant differences were identified for the mono- and polymicrobial infections between five-day and fourteen-day cultures (*p* = 0.135; Table 2). Of the sample, *Staphylococcus aureus* (*S. aureus*) was the most detected organism, found in 34.4% (n = 41) of all positive specimens. Of these, methicillin-resistant *S. aureus* (MRSA) was present in 48.8% (n = 20) of the specimens, and methicillin-sensitive *S. aureus* (MSSA) was present in the remaining 51.2% (n = 21). No significant difference was identified between MRSA and MSSA rates between the five-day and fourteen-day cultures (*p* = 0.541; Table 2). However, the rates pertaining to the different types of organisms detected were significantly different between groups (*p* = 0.016; Table 2). Fungi were only detected in fourteen-day holds (4.5%, n = 2). Five-day holds had higher rates of Gram-negatives (18.7%, n = 14) and anaerobes (1.3%, n = 1). Gram-positives comprised the most common type of organism and were detected in both groups (Table 2).

**Figure 1:**
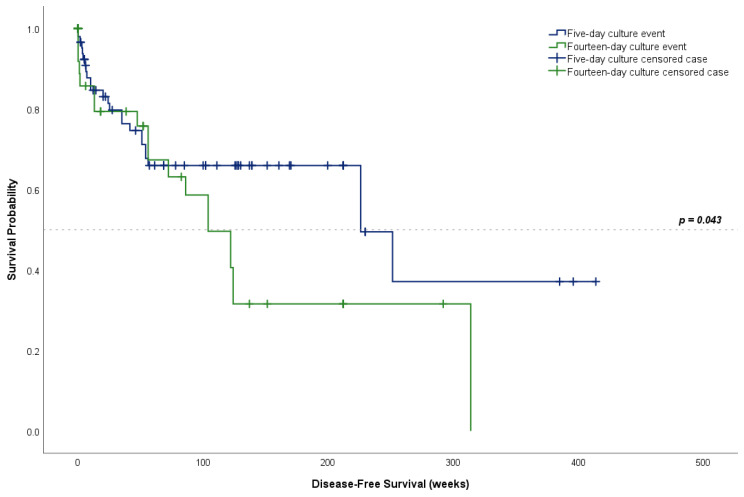
Kaplan–Meier curves for disease-free survival for five-day and fourteen-day culture cases.

The use of postoperative broad-spectrum antibiotics and culture-specific monotherapy was similar between the five-day and fourteen-day cultures (*p* = 0.996); additionally, definitive surgical management was also similar across groups (*p* = 0.196, Table 2). The surgical treatment failure rate was higher for the five-day cultures than for the fourteen-day cultures (74.5%, n = 73 vs. 59.2%, n = 29, respectively, *p* = 0.087), though not significantly different. Of these, 32.4% (n = 33) were reported in culture-negative cases and 67.6% (n = 69) in culture-positive cases. Kaplan–Meier survival curves reported a median disease-free survival of 226.0 weeks (95% CI: 106.9–345.1, Figure 1). The curve comparison using the log-rank test showed a significant difference between the survival curves for five-day and fourteen-day cultures (*p* = 0.043). Also, the median disease-free survival in the five-day cultures was 226.0 weeks (95% CI: 79.2–372.8) and it was 104.0 weeks in the fourteen-day cultures (95% CI: 65.2–142.8).

### 3.2. Institutional Organism Profile

The time to positive culture by organism was significantly different, as *Trichosporon* sp. had the longest median time to positive (i.e., 23 days) and *Finegoldia magna* had the shortest (i.e., 0 days, *p* = 0.011). Subgroup analysis for low-virulence organisms demonstrated no significant difference between *C. acnes*, *C. albicans*, mycobacteria, and anaerobes (*p* = 0.407; Table 3). The time to positive culture by organism type was not statistically significant (*p* = 0.063; Table 3).

The rates of mono- and polymicrobial infections across PJI types were not significantly different (*p* = 0.492; Table 4). Additionally, the types of organism across types of PJI were not significantly different (*p* = 0.312; Table 4). However, surgical treatment failure was reported in 69.4% (n = 102) of cases. The comparison by PJI type was not statistically significant (*p* = 0.107), with chronic PJI in 76.5% (n = 78) of cases, acute hematogenous in 13.7% (n = 14) of cases, and early postoperative in 9.8% (n = 10; Table 5) of cases. When analyzed by the type of organism with mono- and polymicrobial infections, no significant differences were identified across the rates of surgical treatment failure (*p* = 0.394 and *p* = 1.000, respectively; Table 5).

Postoperative complication rates were compared across types of PJI, organisms, and colonization. Deaths during the first 365 days after surgery were reported in 2.0% of cases (n = 3). Comparison by PJI type was not statistically significant (*p* = 1.000), with chronic PJI identified in 66.7% (n = 2) of cases, followed by acute hematogenous in 33.3% (n = 1), and no reported deaths for early postoperative PJIs (Table 5). Similarly, when analyzed by the type of organism and mono- and polymicrobial infections, no significant differences were identified across mortality rates (*p* = 0.080 and *p* = 0.315, respectively, Table 5). Readmissions during the first 30 days after surgery were reported in 22.4% (n = 33) of cases. The comparison by organism type was statistically significant (*p* = 0.050), with Gram-positive organisms identified in 81.0% (n = 17) of cases, followed by Gram-negative organisms (4.8%, n = 1), fungi (4.8%, n = 1), and anaerobes (4.8%, n = 1). However, when analyzed by type of infection, organism, and mono- and polymicrobial infections, no significant differences were identified across readmission rates (*p* = 0.360, *p* = 0.114, and *p* = 1.000, respectively; Table 5).

### 3.3. Association of Synovial and Intraoperative Cultures

Preoperatively, 29.9% (n = 44) of cases had a Synovasure^®^ test administered; of these, 70.5% (n = 31) reported negative organism detection, and the remaining 29.5% (n = 13) reported positive organism detection. However, no significant association existed between the organisms reported in Synovasure^®^ testing and the intraoperative cultures (*p* = 0.234). Only six Synovasure^®^ cases accurately reported the same organism found in intraoperative cultures. The congruently reported microorganisms in both tests were *S. agalactie*, MRSA, MSSA, coagulase-negative *S. aureus*, *S. epidermidis*, and *C. acnes*. The organisms that were reported in the intraoperative cultures but not in the preoperative Synovasure^®^ testing were *E. faecium*, *Bacillus* sp., *Corynebacterium* sp., *E. coli*, *E. faecalis*, *K. pneumoniae*, *S. viridans*, and *C. albicans*.

## 4. Discussion

Our results demonstrated that fourteen-day cultures had a median growth time that was significantly longer and included higher rates of fungi detection than those held for five days. Previous literature has reported similar findings where other slow-growth organisms, like *C. acnes*, were detected during the second week of culture [12,22,23]. However, our findings show a median time to positive culture for *C. acnes* of 8.50 days (Table 3), further supporting the idea that shorter culture times potentially miss the diagnosis of slow-growing organisms. 

Disease-free survival following surgical intervention for PJI was significantly longer for patients in the five-day culture group than in the fourteen-day culture group. However, the interpretation of these findings must consider lead-time bias, as the institutional shift from shorter to longer culture hold times was time-dependent. Patients in the fourteen-day cohort have yet to be followed long enough to deem this difference clinically significant. Our results failed to demonstrate a significant difference between culture hold times for the number of tissue samples sent for culture, the number of positive tissue samples, the rates of MRSA and MSSA, antibiotic coverage, and definitive surgical management of the PJI. Our findings reiterate those of Klement et al., who also failed to find a significant association between an improved surgical success of PJI treatment and more prolonged culture holds [15].

We found the culture positive rate to be similar across hold times, thus not increasing culture yield. This reiterates previous studies looking at similar comparisons [14,15]. However, it is important to note that other researchers have identified up to a 30% increase in positivity rate for longer culture times [12]. Previous literature, however, has reported concerns with the utility of longer culture times and the risk of an increased detection of contaminating organisms [12,14,15]. Although our results support previous findings on similar rates of polymicrobial infections across culture hold times [15], some organisms commonly categorized as contaminants [12,24] were identified in these polymicrobial cultures, primarily in those in the fourteen-day culture group. As such, there continues to be inconsistency regarding prolonged culture, which holds a risk of higher contamination rates as these organisms also have the potential for infection [12,24]. 

The most common organism detected in this sample was *S. aureus*, followed by coagulase-negative *Staphylococcus* species, which is consistent with epidemiological reports of PJI microbiology [13,25,26]. The time to positive culture was significantly different when compared by organism type; the largest difference was between fungi and Gram-negative organisms’ growth time (Table 3). However, this finding was not the case for low virulence organisms; the median growth time identified for this subgroup was 6.14 days. Both of these findings underscore the potential effect of prolonged culture holds. Significant differences in growth time between commonly slow-growing organisms (e.g., fungi) and faster-growing organisms (e.g., Gram-negatives), and the similarity among organisms considered “low virulence” point to more extended culture holds, allowing for sufficient time to detect these organisms in a sample [12,27]. 

Our cohort’s surgical treatment failure rate was higher than other rates reported in the literature [28,29,30]. However, there is inconsistency regarding the definition of treatment failure present in these studies. Most treatment failure cases were in the five-day culture group, with one-third of failures reported in culture-negative cases. These findings underscore the significance of determining the best available culture hold time. In our study, subgroup analyses did not reveal significant differences across PJI types, organism, and colonization types, which is also supported by previous research that has identified no association between the organism and colonization type and failure rates [31].

Finally, no significant association was identified between the organisms reported from intraoperative cultures and Synovasure^®^ cultures, with only six cases accurately reporting the same organism in both cultures. However, Synovasure^®^ testing reported negative culture results in multiple cases where intraoperative cultures reported organisms commonly qualified as contaminants. This descriptive trend in the data highlights the potential for the culture overgrowth of environmental contaminants, a common pitfall in extended bacterial culture sampling [12,24]. However, clinicians must be mindful that common culture contaminants can also overgrow and cause PJI, regardless of their categorization [12]. Thus, the correlation between intraoperative culture, Synovasure^®^ results, and clinical and laboratory findings is imperative when determining diagnosis, management, and antibiotic coverage [10]. Our findings are echoed by a previous systematic review that identified variable concordance between Synovasure^®^ and intraoperative cultures [32]. Previous research in this institution has supported the use of the Synovasure^®^ test’s alpha-defensin levels to have high sensitivity in PJI diagnosis and specificity following two-stage reimplantation [33,34]. However, further research is necessary to evaluate the role of Synovasure^®^ cultures compared to intraoperative cultures. It remains unclear if there is any association between these results and the length of intraoperative culture hold, as this was not the goal of this study.

The results must be interpreted considering the limitations of this study. First, the retrospective nature of this study introduces an opportunity for selection and lead-time bias. This limitation becomes more important for disease-free survival comparisons, with five-day cultures being the standard of care during the first four years of data collection and fourteen-day cultures for the last two years of data collection. Patients who were diagnosed using five-day cultures were inherently followed for a longer period than the fourteen-day culture patients. Second, there was a relatively small sample size of 147 surgical culture samples for analysis. However, given that this study aimed to analyze outcomes from an uncommon, but impactful, complication of total joint arthroplasty, this sample size and methodology are justifiable and comparable to similar studies. Third, there was an unbalanced sample size between five-day and fourteen-day cultures. Although this distribution of samples was expected due to the period analyzed, this imbalance may impact the study’s statistical power. However, in findings where statistical significance was identified to favor the fourteen-day cultures, this 2:1 sample distribution underscores the meaningfulness of these differences. Fourth, as this study was performed at a single institution, this may limit the generalizability of the results. However, surgeries were performed by eight different fellowship-trained arthroplasty surgeons, introducing variability into the dataset and expanding the applicability of the results. Future research should focus on longer culture holds and sensitivity and specificity regarding contaminant organism detection. Similarly, investigation into the cost-effectiveness of longer culture holds and the potential impact on accurate organism detection and PJI management is needed, as it becomes increasingly clear that there continues to be potential benefits in longer culture holds.

## 5. Conclusions

This retrospective cohort study revealed that fourteen-day cultures did not increase the positivity rate, rate of polymicrobial infections, or number of positive specimens reported, and it also identified a similar disease-free survival to five-day cultures. Fourteen-day culture holds demonstrated higher fungi growth rates and a longer time-to-positivization than five-day cultures. More prolonged culture holds may provide a more thorough organism detection for PJI; however, the risk of contaminant over-detection remains. In light of these findings, arthroplasty surgeons should consider increasing intraoperative culture hold times to fourteen days due to the potential for the increased detection of low-virulence organisms known for introducing complexity to managing PJI cases.

## Figures and Tables

**Table 1:** Patient demographics.

	Total N = 147	5-Day Cultures N = 98	14-Day Cultures N = 49	*p*-Value
Age, mean (SD)	62.0 (12.7)	61.5 (12.1)	61.4 (12.7)	0.976
BMI, mean (SD)	30.8 (9.1)	30.2 (8.8)	31.2 (7.6)	0.612
Sex, n (%)				0.216
Female	63 (42.9)	38 (38.8)	25 (51.0)	
Male	84 (57.1)	60 (61.2)	24 (49.0)	
McPherson Host Grade, n (%)				0.249
A	18 (12.2)	15 (15.3)	3 (6.1)	
B	68 (46.3)	45 (45.9)	23 (46.9)	
C	61 (41.5)	38 (38.8)	23 (46.9)	
Active Smoking, n (%)	28 (19.0)	20 (20.4)	8 (16.3)	0.710
DM, n (%)	47 (32.0)	33 (33.7)	14 (28.6)	0.662
Laterality, n (%)				0.600
Left	72 (49.0)	46 (46.9)	26 (53.1)	
Right	75 (51.0)	52 (53.1)	23 (46.9)	
Periprosthetic Joint Infection Type, n (%)				0.976
Early Postoperative	19 (12.9)	13 (13.3)	6 (12.2)	
Acute Hematogenous	23 (15.6)	15 (15.3)	8 (16.3)	
Chronic	105 (71.4)	70 (71.4)	35 (71.4)	

Abbreviations—BMI: body mass index; DM: diabetes mellitus type II.

**Table 2:** Comparison between 5-day and 14-day cultures.

	Total N = 147	5-Day Cultures N = 98	14-Day Cultures N = 49	*p*-Value
Culture Positive Rate, n (%)	99 (67.3)	65 (66.3)	34 (69.4)	0.852
Intraoperative Specimens, Mean (SD)	8.0 (4.2)	8.3 (3.9)	7.6 (4.9)	0.079
Positive Specimens, Mean (SD)	2.5 (1.4)	2.6 (1.5)	2.4 (1.4)	0.527
Time to Positive Culture, Median (Range)	4.0 (0–23)	3.0 (0–7)	5.0 (0–23)	**<0.001**
Colonization, n (%)				0.135
Monomicrobial	82 (82.2)	57 (87.7)	25 (73.5)	
Polymicrobial	17 (17.2)	8 (12.3)	9 (26.5)	
*S. aureus* sensitivities, n (%)				0.541
MRSA	20 (48.8)	16 (53.3)	4 (36.4)	
MSSA	21 (51.2)	14 (46.7)	7 (63.6)	
Organism Type, n (%)				**0.016**
Gram-Positive	100 (84.0)	60 (80.0)	40 (90.9)	
Gram-Negative	16 (13.4)	14 (18.7)	2 (4.5)	
Anaerobe	1 (0.8)	1 (1.3)	0 (0.0)	
Fungi	2 (1.7)	0 (0.0)	2 (4.5)	
Definitive Surgery, n (%)				0.196
DAIR	24 (16.3)	16 (16.3)	8 (16.3)	
1-Stage	33 (22.4)	18 (18.4)	15 (30.6)	
2-Stage	73 (49.7)	49 (50.0)	24 (49.0)	
Resection Arthroplasty	16 (10.9)	14 (14.3)	2 (4.1)	
Amputation	1 (0.7)	1 (1.0)	0 (0.0)	
Antibiotic Spectrum, n (%)				0.724
Narrow	8 (6.7)	6 (7.4)	2 (5.3)	
Broad	111 (93.3)	75 (92.6)	36 (94.7)	
Surgical Treatment Failure, n (%)	102 (69.4)	73 (71.6)	29 (28.4)	0.087

Abbreviations—MRSA: methicillin-resistant *Staphylococcus aureus*; MSSA: methicillin-sensitive *Staphylococcus aureus*; DAIR: debridement, antibiotics, and implant retention. Bolded: statistically significant.

**Table 3:** Comparison between 5-day and 14-day cultures.

	Individual Organism N (%)	Time to Positive Culture Median Days (Range)	*p*-Value
Organism Type (n = 119)			0.063
Gram-Positive	100 (84.0)	4.0 (0–11)	
Gram-Negative	16 (13.4)	3.5 (3–4)	
Anaerobes *	1 (0.8)	0.00 (0)	
Fungi	2 (1.7)	14.5 (6–23)	
Low Virulence Organisms (n = 8)			0.407
*C. acnes*	5 (4.2)	8.5 (2–11)	
*C. albicans*	1 (0.8)	6.0 (6)	
Mycobacteria **	1 (0.8)	7.0 (7)	
Anaerobes *	1 (0.8)	0.0 (0)	

* Only one obligate anaerobe was identified within the sample, *Finegoldia magna*, and was reported as positive in culture on the same day the sample was sent for culture. ** One type of mycobacteria was identified, *Mycobacteria fortuitum*, and *E. coli* was the only Gram-negative organism identified.

**Table 4:** Comparisons by PJI type.

	Total n (%)	Early Postoperative n (%)	Acute Hematogenous n (%)	Chronic n (%)	*p*-Value *
Colonization					0.492
Monomicrobial	82 (82.8)	14 (82.4)	16 (94.1)	52 (80.0)	
Polymicrobial	17 (17.2)	3 (17.6)	1 (5.9)	13 (20.0)	
Organism Type					0.250
Gram-Positive	100 (84.0)	17 (81.0)	11 (94.4)	66 (82.5)	
Gram-Negative	16 (13.4)	2 (9.5)	1 (5.6)	13 (16.3)	
Anaerobes	1 (0.8)	1 (4.8)	0 (0.0)	0 (0.0)	
Fungi	2 (1.7)	1 (4.8)	0 (0.0)	1 (1.3)	

**Table 5:** Complications and surgical treatment failure comparisons across PJI types.

	30-Day Readmissions n (%)	*p*-Value	365-Day Mortality n (%)	*p*-Value	Surgical Treatment Failure n (%)	*p*-Value
Colonization		1.000		0.315		1.000
Monomicrobial	17 (81.0)		1 (50.0)		57 (82.6)	
Polymicrobial	4 (19.0)		1 (50.0)		12 (17.4)	
Organism Type		0.114		0.180		0.394
Gram-Positive	19 (70.4)		2 (50.0)		65 (80.2)	
Gram-Negative	7 (25.9)		2 (50.0)		13 (16.0)	
Anaerobes	0 (0.0)		0 (0.0)		1 (1.2)	
Fungi	1 (3.7)		0 (0.0)		2 (2.5)	
PJI Type		0.360		1.000		0.107
Early Postoperative	3 (9.1)		0 (0.0)		10 (12.9)	
Acute Hematogenous	3 (9.1)		1 (33.3)		14 (13.7)	
Chronic	27 (81.8)		2 (66.7)		78 (76.5)	

## Data Availability

The dataset analyzed during the study are not publicly available per study protocol. De-identified data may be available from the corresponding author with permission from the University of Florida upon reasonable request.
